# Association Between Periodontitis and SARS-CoV-2 Infection Severity: A Cross-Sectional Study in a Turkish Population

**DOI:** 10.3290/j.ohpd.c_2234

**Published:** 2025-08-26

**Authors:** Aliye Akcalı, Aylin Özgen Alpaydın, Muammer Çelik, Bilge Cansu Uzun Saylan, Mehmet Emin Arayıcı, Olivier Huck

**Affiliations:** a Aliye Akcalı Professor, Department of Periodontology, Faculty of Dentistry, Dokuz Eylül University, Izmir, Turkey; Institute of Health Sciences, Department of Dental Biomaterials, Dokuz Eylül University, Izmir, Turkey; Centre for Oral Clinical Research, Barts and The London School of Medicine & Dentistry, Institute of Dentistry, Queen Mary University of London (QMUL), London, UK. Conceptualisation, methodology, analysis, and drafting the manuscript.; b Aylin Özgen Alpaydın Professor, Department of Pulmonology, Dokuz Eylül University, Faculty of Medicine, Izmir, Turkey. Data collection.; c Muammer Çelik Assistant Professor, Department of Infectious Diseases and Clinical Microbiology, Dokuz Eylül University, Faculty of Medicine, İzmir, Turkey. Data collection.; d Bilge Cansu Uzun Saylan Assistant Professor, Department of Periodontology, Faculty of Dentistry, Dokuz Eylül University, Izmir, Turkey. Data collection.; e Mehmet Emin Arayici Assistant Professor, Department of Public Health, Faculty of Medicine, Dokuz Eylül University, Izmir, Turkey; Department of Biostatistics and Medical Informatics, Faculty of Medicine, Dokuz Eylül University, Izmir, Turkey. Data analysis and interpretation.; f Olivier Huck Professor, INSERM, UMR 1260‚ Osteoarticular and Dental Regenerative Nanomedicine‘, Faculté de Médecine, Fédération de Médecine Translationnelle de Strasbourg (FMTS), Strasbourg, France; Faculté de Chirurgie Dentaire, Université de Strasbourg, Strasbourg, France; Pôle de Médecine et de Chirurgie Bucco-Dentaires, Hôpitaux Universitaires de Stras-bourg, Strasbourg, France. Conceptualisation, methodology, analysis, and drafting the manuscript.

**Keywords:** gingivitis, periodontitis, inflammation, virus, SARS-CoV-2

## Abstract

**Purpose:**

The aim of the present study was to evaluate the association between periodontitis and SARS-CoV-2 infection severity in a Turkish population.

**Methods:**

Adult patients attending hospital consultation and testing positive for SARS-CoV-2 infection were consecutively enrolled in this study. Demographic variables, smoking status, COVID-19 symptoms, SpO2 levels, and markers of inflammation (D-Dimer, lymphocytes and white blood cells count, CRP) were recorded. Patients suspected of periodontal disease were evaluated using self-reported questionnaires (OHIP-14, modified CDC/AAP questionnaire). Periodontal screening score (PESS) was calculated from the questionnaire. Univariate and multivariate logistic regression analyses were performed to evaluate the association between COVID-19-associated parameters and periodontitis.

**Results:**

The study included 134 patients diagnosed with COVID-19. Nearly half of the participants were female (n = 68, 50.7%), and the mean age of the patients was 48.7 ± 18.2 years. A statistically significant majority of individuals (69.2%) were asymptomatic, while 22.3% experienced mild symptoms, and 8.5% reported moderate or severe symptoms. Oxygen saturation was found to be higher in asymptomatic patients (96.4 ± 2.8) compared to mild (90.4 ± 5.1) and moderate/severe patients (86.6 ± 8.9) (P <0.001). There was no statistically significant difference concerning OHIP-14 score (P = 0.316), periodontitis (PESS ≥ 5) (P = 0.130), brushing habits (P = 0.901), and frequency of dental visits (P = 0.975) when considering SARS-CoV-2 infection severity. In multivariate logistic regression analysis, it was concluded that male gender (OR = 2.90, 95% CI: 1.04–8.04, P = 0.040), age 55 and above (OR = 5.94, 95% CI: 1.22–28.76, P = 0.026), and smoking (OR = 0.14, 95% CI: 0.02–0.75, P = 0.022) were statistically significant predictors of SARS-CoV-2 infection severity.

**Conclusions:**

Even the association between SARS-CoV-2 infection severity and periodontitis, evaluated through self-reported outcome measures, were weak: male gender, age, and smoking were independent risk factors for SARS-CoV-2 infection severity in this patient cohort. Further research is warranted to explore these associations comprehensively.

Coronavirus 2 of severe acute respiratory syndrome (SARS-CoV-2), a member of the *Coronaviridae* family, is the responsible pathogen of 2019 coronavirus disease (COVID-19). This disease, first identified in Wuhan (China) in December 2019, affected a significant proportion of the worldwide population and led to a high number of deaths according to the World Health Organisation (WHO) (https://www.covid19treatmentguidelines.nih.gov).^7^ Clinical features range from asymptomatic to severe illness and death, and the most observed symptoms are fever, cough, sore throat, headache, fatigue, myalgia, as well as dyspnoea. Loss of taste and smell may also be observed, and in half of the cases, the recovery period may take more than a month.^24^

Periodontitis is an inflammatory disease induced by bacterial dysbiosis. However, several risk factors have been identified, including genetic polymorphisms, lifestyle, systemic conditions and unfavourable local or dental factors.^29,31^ There is a dynamic interaction between periodontitis and systemic diseases, with a particularly significant correlation among endocrinology, immunology, rheumatology, and vascular diseases.^20^ In a recent international consensus report, it was revealed that conditions such as diabetes, obstructive sleep apnoea, chronic obstructive pulmonary disease, and COVID-19 complications are all independently associated with periodontitis.^16^

As periodontitis has been linked to numerous systemic diseases and specifically respiratory diseases,^15,26^ studies were conducted to evaluate the potential association between COVID-19 and periodontitis. Indeed, some studies reported a significant association between periodontitis and SARS-CoV-2 infection or its associated complications.^1,30^ A recent meta-analysis did not measure any direct association between COVID-19 and periodontitis.^26^ However, an association between periodontitis and COVID-19 complications, such as the need for assisted ventilation or mortality, was observed.

The oral cavity is an important reservoir for respiratory pathogens (viruses) and an entryway for the lower respiratory tract.^19^ For instance, periodontal herpes viruses and bacteria can work synergistically to increase the severity of the disease. The contribution of herpes viruses to periodontitis is explained by their induction of proinflammatory cytokines release while suppressing anti-inflammatory cytokines, as well as by reducing the humoral immunity of macrophages and granulocytes against bacterial attacks, thereby promoting bacterial proliferation.^33,37^ In individuals infected with COVID-19, dysbiosis has been detected in oropharyngeal swab samples and saliva due to decreased microbiota diversity. *Neisseria*, one of the dominant species associated with oral health, is a critical member of a healthy commensal microbiome. However, disruptions in the oral microbiome, along with decreased levels of Neisseria during COVID-19 infection, have been linked to various diseases, including periodontal disease.^12^

SARS-CoV-2 has been shown to be found in individuals’ nasopharyngeal, salivary, and gingival sulcus fluids.^14,17^ Therefore, several studies were conducted to evaluate the impact of SARS-CoV-2 on oral tissues and specifically periodontal tissues. Indeed, several biological hypotheses were suggested to explain potential links between COVID-19 and periodontitis, such as the periodontium as an entry point for SARS-CoV-2, as well as a dissemination route via systemic circulation, and promotion of local infection (periodontitis).^10^ It includes the potential translocation of periodontal pathogens and SARS-CoV-2 from the periodontal pocket to the bloodstream, the infection potential of gingival crevicular fluid and the shedding of epithelial cells from the periodontium.^9^ Further hypotheses have also been proposed, including the involvement of shared risk factors and comorbidities, dysregulated immune response or overexpression of SARS-CoV-2 receptors due to micro-aspiration of periodontal pathogens into the lower respiratory tract.^15,16^

This study aimed to evaluate the presence of periodontal disease, sociodemographic characteristics, and smoking habits in individuals infected with COVID-19, assess blood infection levels and morbidity risk, particularly in hospitalised patients, based on symptom severity, and examine the possible associations among these factors.

## MATERIALS AND METHODS

### Study Design and Participants

This cross-sectional study was conducted by the Department of Periodontology, Department of Pulmonology, Department of Infectious Diseases and Clinical Microbiology of the Dokuz Eylül University, Izmir, Türkiye and approved by the Ethics Committee of the Dokuz Eylül University (6830-GOA). Patients attending hospital for a COVID-19 diagnosis or treatment were enrolled on the study between November 2021 and November 2022. The sample size calculation was performed using G*Power 3.1 software. It was planned to reach a total of 128 patients in the t-test family, assuming an unknown frequency with a two-tailed approach, with a medium effect size, the worst acceptable error rate of 5%, with 80% power, and a confidence level of 95%. Patients were enrolled in this study consecutively if they met the following criteria: age ≥ 18 years; tested positive for SARS-CoV-2 using a PCR test. Patients illiterate or unable to understand the objectives of the study were excluded. Written informed consent was obtained from all participants before enrollment in the study.

### Data Collection

Demographic data, including age, gender, education level, working status, body mass index (BMI), and smoking status (yes/no/former), were gathered. Symptoms related to COVID-19 including smell and taste loss, presence of comorbidity, hospitalisation requirement, peripheral oxygen saturation (SpO2), presence of COVID-19-associated pneumonia, vaccination history, blood analysis parameters (D-Dimer (mg/L)), lymphocytes and white blood cells (WBC) counts (10^3^/ μl) and C- reactive protein level (CRP (mg/L)) were collected.^28^

### SARS-CoV-2 Infection Severity Assessment

Severity of SARS-CoV-2 infection was defined as asymptomatic (absence of symptoms), mild (presence of symptoms but without dyspnoea and radiological signs), moderate (clinical or radiological lower track infection, SpO_2_ ≥ 94%) and severe (SpO_2_ <94%, PaO_2_/FiO_2_ <300, respiratory rate >30/ min, or pulmonary involvement >50%).^28^

### Self-Reported Oral and Periodontal Health Assessment

Patients suspected of having periodontal disease were evaluated using a modified version of the CDC/AAP self-reported questionnaire for the surveillance of periodontitis.^5^ Briefly, the questionnaire includes 12 questions on periodontal disease (self-perception of gum health and diseases, history of periodontal treatment, report of tooth mobility, bone loss, current abnormalities related to oral health, gingival bleeding, food impaction, tooth loosening and gum retraction, as well as frequency of interdental cleaning device or mouthrinse use). Then, a periodontal screening score (PESS) was calculated from the questionnaire. Linguistic and cross-cultural adaptation was performed for the Turkish language prior to the study, and the questionnaire was written in Turkish.^3^

Oral Health Impact Profile-14 (OHIP-14) with 14 questions were collected.^27^ Patients were also questioned on their brushing habits and dental visit frequency.

### Data Analysis

All statistical analyses executed in the study were performed employing SPSS (v29.0) (Armonk, NY, USA) and STATA v18 (College Station, TX, USA) package programmes.^18,34^ Compliance of the data with normal distribution was assessed through Kolmogorov–Smirnov and Shapiro–Wilk tests, as well as Skewness and Kurtosis symmetric distributions. Categorical variables are documented as numbers and percentages. Continuous variables that comply with normal distribution are reported with mean and standard deviation (SD), and those that do not comply with normal distribution are reported with median and interquartile range (IQR). BMI was calculated as weight/height*height (kg/m^2^). The five points were taken as the cut-off value for the PESS score, and the study population was evaluated by dividing it into two groups (<5 = no periodontitis; ≥ 5 = has periodontitis). OHIP-14 score was administered as a continuous variable, and analyses were conducted within this framework. The primary endpoint of the present study was the PESS score. Analysis of categorical variables was performed using the Chi-square test. Analysis of continuous variables was carried out using ANOVA and the Kruskal–Wallis test, depending on the suitability of the data for normal distribution. The mild and moderate/severe COVID-19 groups (SARS-CoV-2 infection severity) were consolidated into a single category, resulting in two groups (asymptomatic and mild/moderate/severe). Afterwards, the association between periodontitis (yes or no) and SARS-CoV-2 infection severity was tested using univariate and multivariate binary logistic regression analysis, and the data were presented with odds ratios (OR) and 95% confidence intervals (CI). The evaluation of statistical significance was quantified at the P <0.05 level, and this level was deemed significant in all tests performed.

## RESULTS

### Baseline and Sociodemographic Characteristics of The Population

A total of 134 patients diagnosed with COVID-19 were included in this study. Almost half of the population sample consisted of women (n = 68, 50.7%), and the mean age of the patients was 48.7 ± 18.2 years. It was observed that 24.9% (n = 33) of the individuals included in the study were current smokers, and 15% (n = 20) had a BMI >30. When patients were classified according to SARS-CoV-2 infection severity, 69.2% (n = 90) were asymptomatic, 22.3% (n = 29) presented mild symptoms, and 8.5% (n = 11) presented moderate or severe manifestations. Baseline characteristics and other sociodemographic parameters are summarised in Table 1.

**Table 1:** Sociodemographic and baseline characteristics of the study groups

Parameters	Total (n = 134)
**Age, years, mean ± SD**	48.69 ± 18.31
**Gender, n (%)** Male Female	66 (49.3) 68 (50.7)
**Smoking, n (%)** Never Former Current	69 (51.9) 31 (23.3) 33 (24.8)
**BMI, n (%)** <18.5 18.5–24.9 25–29.9 >30	8 (6.0) 57 (42.9) 48 (36.1) 20 (15.0)
**Education level, n (%)** None Primary school Secondary school High school Bachelor	2 (1.5) 24 (17.9) 9 (6.7) 36 (26.9) 63 (47.0)
**Working, n (%)** No Yes	50 (37.9) 82 (62.1)
**COVID severity, n (%)** Asymptomatic Mild Moderate/severe	90 (69.2) 29 (22.3) 11 (8.5)
**Periodontitis (PESS score), n (%)** No (<5) Yes (≥5)	76 (56.7) 58 (43.3)
SD, standard deviation; BMI, body mass index.

### Medical Findings

COVID-19-related medical parameters were differentiated according to SARS-CoV-2 infection severity and are described in Table 2. The oxygen saturations of the patients included in the study were evaluated according to SARS-CoV-2 infection severity. Accordingly, oxygen saturation was found to be higher in asymptomatic patients (96.4 ± 2.8) compared to mild (90.4 ± 5.1) and moderate/severe patients (86.6 ± 8.9) (P <0.001). Similarly, oxygen saturation in mild (90.4 ± 5.1) patients was found to be statistically significantly higher than in moderate or severe patients (86.6 ± 8.9) (P <0.001). As shown in Table 2, there was no significant difference determined between D-dimer, CRP, WBC, lymphocyte, and SARS-CoV-2 infection severity (P >0.05).

**Table 2:** COVID-19-associated medical parameters of the study groups

Parameters		SARS-CoV-2 infection severity	P value*
Whole group (n = 134)	Asymptomatic (n = 90)	Mild (n = 29)	Moderate/Severe (n = 11)
**SpO2, mean ± SD**	93.3 ± 5.8	96.4 ± 2.8^x^	90.4 ± 5.1^y^	86.6 ± 8.9^z^	**<0.001** ^a^
**D-Dimer, mg/L, median (IQR)**	1.17 (0.6–2.5)	0.74 (0.29–1.40)	1.00 (0.62–24.86)	1.88 (1.08–5.22)	0.181^b^
**CRP, mg/L, median (IQR)**	52 (12.6–122)	21.75 (9.5–154.5)	68.7 (9.1–131)	44.5 (19.6–117.2)	0.769^b^
**WBC, 10^3^/μl, median (IQR)**	5700 (4800–8075)	5350 (3875–6105)	6000 (4270–8700)	6000 (5075–9800)	0.459^b^
**Lymphocyte, 10^3^/μl, median (IQR)**	1100 (750–1300)	1150 (775–1425)	1100 (682–1200)	1150 (600–2362)	0.458^b^
**Presence of comorbidity, n (%)** No Yes	73 (54.5) 61 (45.5)	59 (65.6) 31 (34.4)	11 (37.9) 18 (62.1)	3 (27.3) 8 (72.7)	**0.004** ^c^
**Hospitalization requirement, n (%)** No Yes	95 (72) 37 (28)	84 (95.5) 4 (4.5)	9 (31.0) 20 (69.0)	1 (9.1) 10 (90.9)	**<0.001** ^c^
**ICU requirement, n (%)** No Yes	120 (93) 9 (7)	90 (100) 0 (0)	24 (100) 0 (0)	2 (18.2) 9 (81.8)	**<0.001** ^c^
**Presence of COVID-19 pneumonia, n (%)** No Yes	85 (64.4) 47 (35.6)	77 (86.5) 12 (13.5)	6 (21.4) 22 (78.6)	1 (9.1) 10 (90.9)	**<0.001** ^c^
**Vaccination history, n (%)** None 1 time 2 times 3 times 4 times	24 (21.6) 12 (10.8) 13 (11.7) 15 (13.5) 47 (42.3)	12 (15.2) 10 (12.7) 12 (15.2) 10 (12.7) 35 (44.3)	8 (34.8) 0 (0) 0 (0) 5 (21.7) 10 (43.5)	2 (33.3) 1 (16.7) 1 (16.7) 0 (0) 2 (33.3)	N/A
SD standard deviation, IQR interquartile range, N/A not available (The test lacks validity as a result of an excessive number of groups), ^a^ ANOVA (LSD posthoc test), ^b^ Kruskal–Wallis test, ^c^ Chi-Square (χ^²^) test, * The ones indicated in bold exhibit statistical significance at the P <0.05 threshold. ^x,y,z^ There was no significant difference between the same letters, and there was a significant difference between separate letters.

The presence of comorbidities such as hypertension, diabetes mellitus, chronic obstructive pulmonary disease, haematological diseases, malignancies, or immune suppressive conditions, the requirement for hospitalisation, the requirement for an ICU, and the presence of COVID-19 pneumonia in the patients were also scrutinised according to the severity of COVID-19. It has been observed that COVID-19 was more severe in patients with comorbidities (P = 0.004). Data on comparison of SARS-CoV-2 infection severity and other relevant parameters are available in Table 2.

COVID-19-related medical parameters were investigated in groups with (PESS <5) and without periodontitis (PESS ≥ 5). Accordingly, WBC was found to be statistically significantly higher in the periodontitis group [median: 6000 (IQR: 5050-10900) vs median: 5000 (IQR: 2950-6400)] (P = 0.016). No statistically significant difference was observed in terms of oxygen saturation, D-dimer, CRP and lymphocyte (P >0.05).

#### Demographic data and oral health assessment according to SARS-CoV-2 infection severity

**Fig 1:**
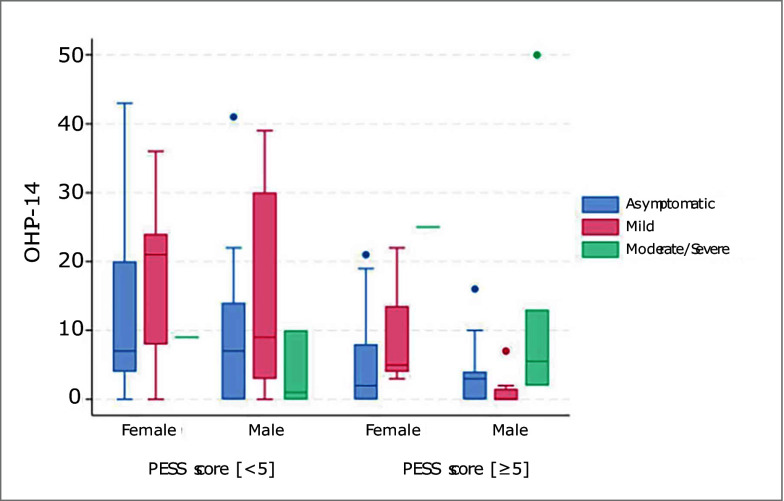
OHIP-14 scores of the participants according to the SARS-CoV-2 infection severity.

The oral health assessment and demographic variables according to SARS-CoV-2 infection severity are described in Table 3, and the self-report questionnaire for the screening and surveillance of periodontal diseases and the OHIP-14 scores of the participants included in the study are reported in Tables 4 and 5. The median OHIP-14 score was 4 (IQR: 0-13). OHIP-14 scores separated by SARS-CoV-2 infection severity groups are illustrated in Figure 1. It was documented that only 50.8% of the study group visited the dentist at least once a year. Regarding their oral hygiene habits, 27.1% declared brushing their teeth twice a week or less.

**Table 3:** The distribution of demographic and periodontal disease information within the study cohort based on the severity of SARS-CoV-2 infection

Parameters		SARS-CoV-2 infection severity	P value*
Whole group (n = 134)	Asymptomatic (n = 90)	Mild (n = 29)	Moderate/Severe (n = 11)
**Age, mean ± SD**	48.69 ± 18.31	43.56 ± 16.90 ^x^	60.38 ± 15.58^y^	55.64 ± 22.07^y^	**<0.001** ^a^
**BMI, mean ± SD**	25.56 ± 5.15	24.47 ± 4.75^x^	27.48 ± 5.25^y^	27.03 ± 3.61^x,y^	**0.008** ^a^
**OHIP-14, median (IQR)**	4 (0–13)	4 (0–9.25)	5 (0–21)	8 (2–13)	**0.316** ^b^
**Gender, n (%)** Female Male	66 (49.3) 68 (50.7)	54 (60) 36 (40)	10 (34.5) 19 (65.5)	2 (18.2) 9 (81.8)	**0.004** ^c^
**Smoking, n (%)** Never Former Current	69 (51.9) 31 (23.3) 33 (24.8)	45 (50.6) 29 (32.6) 15 (16.9)	18 (62.1) 1 (3.4) 10 (34.5)	5 (45.5) 1 (9.1) 5 (45.5)	**0.005** ^c^
**Periodontitis, n (%)** No Yes	76 (56.7) 58 (43.3)	56 (62.2) 34 (37.8)	12 (41.4) 17 (58.6)	7 (63.6) 4 (36.4)	0.130^c^
Brushing habits, n (%)** None or once/twice a week More than one in a day	36 (27.1) 97 (72.9)	22 (24.4) 68 (75.6)	8 (28.6) 20 (71.4)	3 (27.3) 8 (72.7)	0.901^c^
**Dental visit frequency n (%)** 0 >1	60 (49.2) 62 (50.8)	41 (48.2) 44 (51.8)	12 (46.2) 14 (53.8)	4 (50.0) 4 (50.0)	0.975^c^
SD standard deviation, IQR interquartile range,), ^a^ ANOVA (LSD posthoc test), ^b^ Kruskal–Wallis test, ^c^ Chi-Square (χ^2^) test, * The ones indicated in bold exhibit statistical significance at the P <0.05 threshold, ^x,y^ There was no significant difference between the same letters, and there was a significant difference between separate letters. ** The two groups (‘None’ and ‘once/twice a week’) were combined since the number in the non-brushing group was low.

**Table 4:** Self-reported questionnaire for the screening and surveillance of periodontal diseases

Questionnaire items	SARS-CoV-2 infection severity
Asymptomatic (%/n)	Mild (%/n)	Moderate/Severe (%/n)	Whole population (%/n)
**Q1. Do you think you might have gum disease?** • Yes • No • Don’t known • Refused	34.4/31 58.9/53 6.7/6 0	24.1/7 65.5/19 6.9/2 3.4/1	0/0 63.6/7 36.4/4 0/0	29.2 /38 60.8 /79 9.2 /12 0.8/1
**Q2. Overall, how would you rate the health of your teeth and gums?** • Excellent • Very good • Good • Fair • Poor • Don’t know • Refused	2.2/2 14.4/13 38.9/35 40/36 3.3/3 1.1/1 0/0	3.4/1 10.3/3 27.6/8 44.8/13 6.9/2 3.4/1 3.4/1	9.1/1 9.1/1 36.4/4 45.5/5 0/0 0/0 0/0	3.1/4 13.1/17 36.2/47 41.5/54 3.8/5 1.5/2 0.8/1
**Q3. Have you ever had any treatment for gum disease such as scaling and root planning, sometimes called ‘deep cleaning’?** • Yes • No • Don’t known • Refused	28.1/25 67.4/60 4.5/4 0/0	31.0/9 55.2/16 6.9/2 3.4/1	27.8/3 72.7/8 0/0 0/0	28.9/37 65.6/84 4.7/6 0.8/1
**Q4. Have you ever had any teeth become loose on their own, without an injury?** • Yes • No • Don’t known • Refused	14.6/13 80.9/72 3.4/3 1.1/1	34.5/10 51.7/15 10.3/3 3.4/1	27.3/3 54.5/6 9.1/1 0/0	20.3/26 72.7/93 5.5/7 1.6/2
**Q5. Have you ever been told by a dental professional that you lost bone around your teeth?** • Yes • No • Don’t known • Refused	10/9 88.9/80 1.1/1 0/0	10.3/3 79.3/23 6.9/2 3.4/1	0/0 81.8/9 18.2/2 0/0	9.2/12 86.2/112 3.8/5 0.8/1
**Q6. During the past 3 months, have you noticed a tooth that doesn’t look right?** • Yes • No • Don’t known • Refused	41.6/37 58.4/52 0/0 0/0	20.7/6 62.1/18 3.4/1 6.9/2	0/0 63.6/7 9.1/1 9.1/1	34.4/43 61.6/77 1.6/2 2.4/3
**Q7. Aside from brushing your teeth with a toothbrush, in the last seven days, how many times did you use dental floss or any other device to clean between your teeth?** Mean number of days • Never • Once or more • Refused	28.9/26 66.7/60 4.4/4	6.9/2 89.7/26 3.4/1	9.1/1 81.8/9 9.1/1	22.3/29 73.1/95 4.6/6
**Q8. Aside from brushing your teeth with a toothbrush, in the last 7 days, how many times did you use mouthwash or other dental rinse product that you use to treat dental disease or dental problems?** **Mean number of days** • Never • Once or more • Refused	25.6/23 66.7/60 7.8/7	6.9/2 89.7/26 3.4/1	9.1/1 90.9/10 0/0	19.9/26 73.9/96 6.2/8
**Q9. Have your gums bled recently?** • Yes • No • Don’t known • Refused	32.2/29 66.7/60 1.1/1 0/0	17.2/5 79.3/23 0/0 3.4/1	9.1/1 54.5/6 9.1/1 0/0	27.6/35 70.1 /89 1.6/2 0.8/1
**Q10. Do you have food impaction between your teeth?** • Yes • No • Don’t known	67.4/60 32.6/29 0/0	69.0/20 31.0/9 0/0	87.5/7 0/0 12.5/1	69/87 30.2/38 0.8/1
**Q11. Do you notice your teeth getting longer?** • Yes • No • Don’t known • Refused	17.8/16 77.8/70 4.4/4 0/0	13.8/4 75.9/22 3.4/1 6.9/2	0/0 71.4/5 28.6/2 0/0	15.9/20 77/97 5.6/7 1.6/2
**Q12. Do you think that you can see more roots of teeth than in the past?** • Yes • No • Don’t known • Refused	25.6/23 70/63 4.4/4 0/0	24.1/7 58.6/17 13.8/4 3.4/1	0/0 62.5/5 37.5/3 0/0	23.6/30 66.9/85 8.7/11 0.8/1


**Table 5:** OHIP-14 scores of the participants included in the study

Items	SARS-CoV-2 infection severity
Because of problems with limitation your teeth, mouth or dentures:	Asymptomatic (%/n)	Mild (%/n)	Moderate/Severe (%/n)	Whole population (%/n)
**1) Have you had trouble pronouncing any words?** Never Hardly ever Occasionally Fairly often Very often	83.1/74 7.9/7 6.7/6 1.1/1 1.1/1	60.7/17 25.0/7 7.1/2 0/0 7.1/2	63.6/7 9.1/1 18.2/2 0/0 9.1/1	76.6/98 11.7/15 7.8/10 0.8/1 0.8/1
**2) Have you felt that your sense of taste has worsened?** Never Hardly ever Occasionally Fairly often Very often	72.4/63 11.5/10 11.5/10 1.1/1 3.4/3	75.0/21 14.3/4 3.6/1 3.6/1 3.6/1	45.5/5 18.2/2 27.3/3 0/0 9.1/1	70.6/89 12.7/16 11.1/14 1.6/2 4/5
**3) Have you had painful aching in your mouth?** Never Hardly ever Occasionally Fairly often Very often	51.1/45 19.3/17 19.3/17 8/7 2.3/2	53.6/15 21.4/6 17.9/5 3.6/1 3.6/1	63.6/7 18.2/2 9.1/1 0/0 9.1/1	52.8/67 19.5/25 18.1/23 6.3 /8 3.1/4
**4) Have you found it uncomfortable to eat any foods?** Never Hardly ever Occasionally Fairly often Very often	57.3/51 12.4/11 18/16 9/8 3.4/3	53.6/15 10.7/3 21.4/6 3.6/1 10.7/3	63.6/7 18.2/2 9.1/1 0/0 9.1/1	57/73 12.5/16 18/23 7/9 5.5/7
**5) Have you been self-conscious?** Never Hardly ever Occasionally Fairly often Very often	76.1/67 10.2/9 6.8/6 4.5/4 2.3/2	71.4/20 7.1/2 7.1/2 10.7/3 3.6/1	72.7/8 18.2/2 0/0 0/0 9.1/1	74.8/95 10.2/13 6.3/8 5.5/7 3.1/4
**6) Have you felt tense?** Never Hardly ever Occasionally Fairly often Very often	71.6/63 12.5/11 6.8/6 6.8/6 2.3/2	51.9/14 11.1/3 11.1/3 11.1/3 14.8/4	54.5/6 18.2/2 18.2/2 0/0 9.1/1	65.9/83 12.7/16 12.7/16 7.1/9 5.6/7
**7) Has your diet been unsatisfactory?** Never Hardly ever Occasionally Fairly often Very often	77/67 12.6/11 4.6/4 3.4/3 2.3/2	57.1/16 10.7/3 10.7/3 10.7/3 10.7/3	72.7/8 0/0 9.1/1 9.1/1 9.1/1	72.2/91 11.1/14 6.3/8 5.6/7 4.8/6
**8) Have you had to interrupt meals?** Never Hardly ever Occasionally Fairly often Very often	75/66 12.5/11 4..5/4 4.5/4 3.4/3	67.9/19 14.3/4 10.7/3 3.6/1 3.6/1	81.8/9 0/0 0/0 9.1/1 9.1/1	74/94 11.8/15 5.5/7 4.7/6 3.9/5
**9) Have you found it difficult to relax?** Never Hardly ever Occasionally Fairly often Very often	64/57 13.5/12 14.6/13 3.4/3 4.5/4	60.7/17 3.6/1 10.7/3 17.9/5 7.1/2	63.6/7 9.1/1 18.2/2 9.1/1 0/0	63.3/81 10.9/14 14.1/18 7/9 4.7/6
**10) Have you been a bit embarrassed?** Never Hardly ever Occasionally Fairly often Very often	79.5/70 10.2/9 8/7 1.1/1 1.1/1	67.9/19 17.9/5 3.6/1 7.1/2 3.6/1	54.5/6 9.1/1 27.3/3 0/0 9.1/1	74.8 /95 11.8/15 8.7/11 2.4/3 2.4/3
**11) Have you been a bit irritable with other people?** Never Hardly ever Occasionally Fairly often Very often	80.7/71 10.2/9 5.7/5 2.3/2 1.1/1	60.7/17 17.9/5 7.1/2 7.1/2 7.1/2	63.6/7 9.1/1 18.2/2 0/0 9.1/1	74.8/95 11.8 /15 7.1/9 3.1/4 3.1/4
**12) Have you had difficulty doing your usual jobs?** Never Hardly ever Occasionally Fairly often Very often	79.5/70 11.4/10 8/7 0/0 1.1/1	60.7/17 14.3/4 21.4/6 0/0 3.6/1	60.0/6 0/0 10.0/1 20.0/2 10.0/1	73.8/93 11.1/14 11.1/14 1.6/2 2.4/3
**13) Have you felt that life in general was less satisfying?** Never Hardly ever Occasionally Fairly often Very often	68.2/60 17 /15 12.5/11 1.1/1 1.1/1	64.3/18 13.3/4 3.6/1 14.3/4 3.6/1	54.5/6 18.2/2 27.3/3 0/0 0/0	66.1/84 16.5/21 11.8/15 3.9/5 1.6/2
**14) Have you been totally unable to function?** Never Hardly ever Occasionally Fairly often Very often	90.9/80 4.5/4 2.3/2 0/0 2.3/2	75.0/21 0/0 14.3/4 7.1/2 3.6/1	70.0/7 20.0/2 0/0 0/0 10.0/1	85.7/108 4.8/6 4.8/6 1.6/2 3.2/4


The distribution of demographic data according to SARS-CoV-2 infection severity was assessed. Accordingly, the mean age in the asymptomatic group was found to be statistically significantly lower than in the mild and moderate/severe groups (P <0.001). When BMI was examined, it was sstatistically significantly lower in the asymptomatic group than in the mild group (P = 0.008). There was no significant difference between other groups (P >0.05). Additionally, SARS-CoV-2 infection severity was statistically significantly higher in men than in women (P = 0.004) (Table 3). No statistically significant difference was observed when SARS-CoV-2 infection severity was evaluated according to the OHIP-14 score (p = 0.316), periodontitis (P = 0.130), brushing habits (P = 0.901), and dental visits (P = 0.975) (Table 3).

#### Regression analyses associated with SARS-CoV-2 infection severity and various parameters

Mild and moderate/severe groups were combined under one roof, and the cohort was divided into two groups (asymptomatic, mild/moderate/severe), and univariate and multivariate regression analyses were carried out to investigate the impact of various parameters on SARS-CoV-2 infection severity (Table 6). Accordingly, in univariate analysis, male gender (OR = 3.50, 95% CI: 1.57–7.76, P = 0.002), OHIP14 score (OR = 1.03, 95% CI: 1.00–1.07, P = 0.040), BMI (OR = 1.12, 95% CI: 1.04–1.22, P = 0.004), age 55 and above (OR = 7.01, 95% CI: 2.68–18.33, P <0.001) and presence of comorbidity (OR = 3.53, 95% CI: 1.61–7.72, P = 0.002), and current smoking (OR = 0.13, 95% CI: 0.02–0.61, P = 0.009) were identified as independent risk factors for SARS-CoV-2 infection severity. In multivariate logistic regression analysis, it was concluded that male gender (OR = 2.90, 95% CI: 1.04–8.04, P = 0.040), age 55 and above (OR = 5.94, 95% CI: 1.22–28.76, P = 0.026), and smoking (OR = 0.14, 95% CI: 0.02–0.75, P = 0.022) werestatistically significant predictors of SARS-CoV-2 infection severity.

**Table 6:** Univariate and multivariate regression analyses associated with SARS-CoV-2 infection severity and various parameters

Parameter	Participants, n (%)	Univariate	Multivariate
OR	[95% CI]	P value	OR	95% CI	P value
**OHIP-14 score**	134 (100)	1.03	1.00–1.07	0.04	1.04	0.98–1.10	0.144
**BMI (kg/m2)**	133 (100)	1.12	1.04–1.22	0.004	1.07	0.97–1.19	0.140
**Gender** Female (reference) Male	68 (50.7) 66 (49.3)	3.50	1.57–7.76	**0.002**	2.90	1.04–8.04	**0.040**
**Age group** 19–39 (reference) 40–54 55 and above	49 (36.6) 37 (27.6) 48 (35.8)	1.02 7.01	0.32–3.26 2.68–18.33	0.966 **<0.001**	1.01 5.94	0.26–3.82 1.22–28.76	0.985 **0.026**
**PESS score** No (<5) (reference) Yes (≥ 5)	76 (56.7) 58 (43.3)	1.82	0.85–3.86	0.118	0.47	0.12–1.76	0.264
**Comorbidity** No (reference) Yes	73 (54.5) 61 (45.5)	3.53	1.61–7.72	**0.001**	1.53	0.50–4.72	0.450
**Smoking** Never (reference) Current Former	69 (51.9) 31 (23.3) 33 (24.8)	0.13 1.95	0.02–0.61 0.81–4.69	**0.009** 0.132	0.14 0.58	0.02–0.75 0.17–1.93	**0.022** 0.377
OR, odds ratio; CI, confidence interval; PESS, periodontal screening score; OHIP-14, oral health impact profile-14; BMI, body mass index.

## DISCUSSION

The present study showed that, in a Turkish population, the severity of COVID-19 manifestations was linked to impaired oral health. Indeed, it confirms the potential link between oral health and COVID-19.

The burden of COVID-19 challenged health systems in all countries due to its consequences in terms of hospitalisation rates and long-term health effects.^25^ Several strategies have been developed to reduce the risk of developing a severe form of the disease, including immunisation and risk factor management.^11^ Amongst potential risk factors, oral health and periodontal conditions have been suggested.^9^ Recently, a meta-analysis showed that patients with periodontitis are more likely to experience a more severe course of COVID-19.^2^ More interestingly, such patients were more at risk of hospitalisation (OR = 4.72), requiring assisted ventilation (OR = 6.24) and death (OR = 7.51). In the included Turkish population, male gender (OR = 2.90), age 55 and above (OR = 5.94), smoking (OR = 0.14) and BMI (OR = 1.10) were identified as independent risk factors for SARS-CoV-2 infection severity. These results are in accordance with other epidemiological studies performed, highlighting notably the link between obesity and gender and increased SARS-CoV-2 infection severity.^13,23^

In this study, PESS was used to determine the presence of severe cases of periodontitis in the studied population. PESS is based on the CDC/AAP 8-item self-reported measure to which four additional items were added.^5^ Such a score demonstrated good accuracy, moderate sensitivity, and specificity when tested in a French population. Such a tool is of interest to screen periodontal status and to determine the most severe cases, characterised by the need for further complex treatments. Here, 43% of the whole population presented a PESS≥5 and could be considered as presenting a severe case of periodontitis. Such prevalence of severe periodontitis could be considered high when compared to recent epidemiological data.^22^ Interestingly, no statistically significant association was measured between severe periodontitis (PESS ≥5) and SARS-CoV-2 infection severity. This result could be explained by the sample size. However, it is important to note that multivariable analysis showed that PESS was associated with SARS-CoV-2 infection severity, strengthening the hypothesis of a link between COVID-19 and periodontitis severity.

It has been reported that oral microbiota dysbiosis or certain oral pathogens associated with periodontitis^6^ may play a role in COVID-19 infection and disease severity.^35^ It is stated that the oral microbiota may influence SARS-CoV-2 infection through its interaction with host cells and that pathogenic bacteria may exacerbate SARS-CoV-2 infection by promoting the production of proinflammatory cytokines such as IL-6, IL-23, and IL-1.32. The oral microbiota has previously been shown to modulate both innate and adaptive immunity, which may contribute to the progression of SARS-CoV-2 infection. Additionally, it has been suggested that in individuals with respiratory diseases such as pneumonia and chronic obstructive pulmonary disease, the translocation of periodontal pathogens to the lungs may facilitate the replication of SARS-CoV-2 in lung cells.^21^ It has been observed that periodontopathogens such as *Porphyromonas gingivalis*, which reside in deep periodontal pockets, may increase the risk of complications in SARS-CoV-2 infection. Furthermore, studies have shown that *P. gingivalis* lipopolysaccharides can induce the expression of the SARS-CoV-2 entry receptor ACE2 and the accessory protease TMPRSS2 in human gingival fibroblasts. This mechanism is considered a potential pathway explaining why COVID-19 infection may be more severe in patients with periodontitis.^4^

The COVID-19 pandemic has had a deleterious impact on oral health, notably due to its influence on long-term patient follow-ups. In China, a retrospective study observed that the COVID-19 pandemic was an important factor explaining the increased loss to follow-up of patients included in a supportive periodontal treatment programme.^36^ Same trend was also observed in Japan, where intervals between SPT were extended, leading to periodontal parameters worsening.^38^

Moreover, it was also observed that during this particular period, preventive procedures provided in the adult population were statistically significantly lower than those observed in the USA.8 In the studied population, only half benefited from a recent visit to the dentist (<1 year), and the consideration of oral hygiene was low, as only 27% of the individuals declared to brush their teeth two times/week or less. It cannot be argued that this lack of dental check-up in this population was influenced by the COVID-19 pandemic due to the cross-sectional design of the study; however, such a parameter should be considered while interpreting the data.

Nevertheless, this study presents some limitations that should be addressed in future studies. The study’s limitations include the small sample size and the inability to perform periodontal examinations directly on COVID-19-positive individuals. Indeed, to better determine the impact of the extent and severity of periodontitis, future studies should implement a systematic clinical diagnosis based on periodontal charting and radiographic analysis. Moreover, as inflammatory burden has been considered as a potential biological link between periodontitis and COVID-19, as well as for other respiratory diseases, the evaluation of the inflammatory status of the periodontium would be of interest through, for instance, the evaluation of the periodontal inflamed surface area. Moreover, precise periodontal diagnosis using current classification should be considered to distinguish treated and untreated periodontal cases and to determine the influence of distribution, severity, complexity and rate of progression on COVID-19 manifestations.

## CONCLUSION

In a Turkish population affected by COVID-19, this study demonstrated that oral health should be considered as a risk factor of SARS-CoV-2 infection severity. A specific emphasis should be made to control and treat oral diseases, especially periodontitis, to reduce the risk of major complications linked to COVID-19, notably in a population exhibiting other comorbidities. Future studies should focus on the impact of periodontitis management on COVID-19 prevention and the rate of complications to prevent mortality.
